# Genetic dissection of reproductive performance of dairy cows under heat stress

**DOI:** 10.1111/age.12943

**Published:** 2020-05-03

**Authors:** A. Sigdel, L. Liu, R. Abdollahi‐Arpanahi, I. Aguilar, F. Peñagaricano

**Affiliations:** ^1^ Department of Animal Sciences University of Florida Gainesville FL 32611 USA; ^2^ Instituto Nacional de Investigación Agropecuaria Montevideo 11100 Uruguay; ^3^ University of Florida Genetics Institute University of Florida Gainesville FL 32611 USA

**Keywords:** cow conception rate, pathway analysis, thermotolerance, whole‐genome scan

## Abstract

Heat stress negatively impacts the reproductive performance of dairy cows. The main objective of this study was to dissect the genetic basis underlying dairy cow fertility under heat stress conditions. Our first goal was to estimate genetic components of cow conception across lactations considering heat stress. Our second goal was to reveal individual genes and functional gene‐sets that explain a cow’s ability to conceive under thermal stress. Data consisted of 74 221 insemination records on 13 704 Holstein cows. Multitrait linear repeatability test‐day models with random regressions on a function of temperature–humidity index values were used for the analyses. Heritability estimates for cow conception under heat stress were around 2–3%, whereas genetic correlations between general and thermotolerance additive genetic effects were negative and ranged between −0.35 and −0.82, indicating an unfavorable relationship between cows’ ability to conceive under thermo‐neutral vs. thermo‐stress conditions. Whole‐genome scans identified at least six genomic regions on BTA1, BTA10, BTA11, BTA17, BTA21 and BTA23 associated with conception under thermal stress. These regions harbor candidate genes such as *BRWD1*, *EXD2*,* ADAM20*,* EPAS1*,* TAOK3*, and *NOS1*, which are directly implicated in reproductive functions and cellular response to heat stress. The gene‐set enrichment analysis revealed functional terms related to fertilization, developmental biology, heat shock proteins and oxidative stress, among others. Overall, our findings contribute to a better understanding of the genetics underlying the reproductive performance of dairy cattle under heat stress conditions and point out novel genomic strategies for improving thermotolerance and fertility via marker‐assisted breeding.

## Introduction

Heat stress negatively impacts dairy cattle performance. It is now known that the intense selection for productive traits in recent decades has compromised the thermoregulatory competence of dairy cows (Aguilar *et al.* 2009; Santana *et al.* 2017; Sigdel *et al*. 2019). In particular, heat stress is a major cause of low fertility in dairy cattle. Indeed, heat stress disrupts several reproductive processes, including follicular growth, steroid production, estrous expression, oocyte competence, uterine endometrial response and embryonic growth, leading to conception failure, early embryonic mortality and pregnancy loss (Jordan 2003; Hansen 2009; Silva *et al.* 2013). Several studies have reported that there is a marked decrease in pregnancy rate during the summer (Hansen & Arechiga 1999; Sartori *et al.* 2002; de Vries & Risco 2005). Summer heat stress affects reproductive performance because cows undergo adaptive mechanisms during periods of thermal stress by redirecting blood flow from core to periphery, thereby impacting cyclicity, pregnancy establishment and fetal development (Al‐Katanani *et al.* 2002; Hansen 2009). In dairy herds in the southeast USA, there is a strong seasonal variation in conception per insemination. For instance, de Vries & Risco (2005) investigated the seasonality of annual pregnancy rate (between 71 and 364 days since last calving) in dairy herds in Florida and Georgia, reporting a 15.8% pregnancy rate during winter compared with 5.6% during summer. This decline in pregnancy rate during periods of heat stress has a huge economic cost to the dairy industry (De Vries 2006). Various strategies, including cooling, shading and nutrition, have been used to alleviate the negative effects of thermal stress on dairy cow fertility. Nonetheless, there is a clear decline in conception in hot and tropical climates (Schuller *et al.* 2014; Hagiya *et al.* 2017). In this context, genetic selection for improved thermotolerance is an attractive complementary approach for reducing the effects of heat stress and subsequently improving reproductive performance.

Ravagnolo & Misztal (2000) presented a methodology for genetic evaluation of productive and reproductive traits under heat stress by combining test day records with public weather station records. The statistical model has two genetic effects, a regular genetic effect corresponding to performance under thermo‐neutral conditions and a heat‐stress effect corresponding to the rate of decline under thermal stress. Heat stress is measured using a temperature–humidity index (THI, NOAA 1976). In this context, a linear regression of phenotypic records on an environmental variable (THI) is fitted with the assumption that the phenotype is unaffected until a certain threshold level of THI, and above that level, the phenotypic performance declines linearly with increasing THI. Genetic variation is associated with the rate of decline under thermal stress conditions. Using this methodology, Ravagnolo & Misztal (2002) evaluated the effect of heat stress on non‐return rate in US Holstein cows. Recently, using a similar approach, Ansari‐Mahyari *et al. *(2019) investigated the genetic variability of conception rate and also non‐return rate in Iranian dairy cows under heat stress conditions. Both studies indicated that there is a substantial genetic variation underlying conception rate under heat stress, and hence, dairy cow reproductive performance under thermal stress conditions could be genetically improved.

There is increasing knowledge about genes and pathways associated with conception in dairy cattle. For instance, Hoglund *et al. *(2015) identified genomic regions on BTA20 and BTA23 significantly associated with pregnancy rate in Danish Jersey cattle. Liu *et al*. (2017) identified individual genes on BTA8, BTA13 and BTA23, such as *PRPF4B* and *PXDC1*, as potential candidate genes affecting pregnancy establishment and maintenance in Chinese Holstein cattle. Recently, Kiser *et al. *(2019) reported a list of loci, positional candidate genes, including *ARVCF*, *GJB4*, *GJB5* and *JMY*, and transcription factor binding sites associated with conception at first service and number of services per conception in US primiparous Holstein cows. On the other hand, there is no information regarding individual genes, biological pathways and molecular mechanisms affecting dairy cows’ ability to successfully conceive under thermal stress. As such, the first objective of this study was to estimate genetic components for conception per insemination across lactations considering heat stress using random regressions as a function of THI values. The second objective of this study was to perform whole‐genome scans and subsequent gene‐set enrichment analyses in order to identify genomic regions, individual genes and functional gene‐sets implicated in cow conception under heat stress. This relevant information could contribute to improving dairy cows’ ability to conceive under heat stress using novel genomic strategies.

## Materials and methods

### Phenotypic and genotypic data

Conception per insemination is an important fertility trait in dairy cattle, which is defined as the cow’s ability to establish and maintain a successful pregnancy, provided the cow is inseminated at the time of ovulation (Averill *et al.* 2004). Phenotypic data consisted of 74 221 insemination records on 13 704 Holstein cows calved from 2006 to 2016 in two dairy herds in the State of Florida, USA. Service data includes 28 400 insemination records for first‐lactation cows, 26 809 records for second‐lactation cows and 19 012 records for third‐lactation cows. A total of 6838 cows had insemination records in at least two consecutive lactations. All insemination records until 400 days in milk (DIM) were used in the analyses. The outcome of each insemination was recorded as 0 and 1, where 1 indicates that insemination achieved a successful conception and 0 indicates otherwise. The pedigree file included 28 620 animals based on a five‐generation pedigree.

Genotype data for 60 671 SNP markers were available for a total of 4700 cows with insemination records and also 1592 sires in the pedigree. The SNP data were kindly provided by the Cooperative Dairy DNA Repository and the Council on Dairy Cattle Breeding. Those SNP markers that mapped to sex chromosomes, were monomorphic or had minor allele frequency less than 1% were removed from the genotype data. After quality control, a total of 58 046 SNPs were retained for whole‐genome scans and subsequent gene‐set analyses.

### Weather information

Weather data were obtained from Florida Automated Weather Network for Alachua county (https://fawn.ifas.ufl.edu/) and hourly THI was calculated following Bohmanova *et al. *(2007) as THI=(1.8temp+32)-(0.55-0.0055rh)(1.8temp-26), where ‘temp’ is the temperature in degrees Celsius and ‘rh’ is the relative humidity as a percentage. Mean daily THI of day of insemination was assigned to each insemination record as suggested by Ravagnolo & Misztal (2002).

A function of THI, denoted as *f*(THI), was created as a dummy variable in order to estimate decline in reproductive performance under heat stress conditions:

$$f\left(\text{THI}\right)=\left\{\begin{array}{cc} 0 & \text{if}\;\text{THI}\leq {\text{THI}}_{\text{thr}}\\ \text{THI}-{\text{THI}}_{\text{thr}\;} & \text{if}\;\text{THI}>{\text{THI}}_{\text{thr}} \end{array}\right.,$$

where THI_thr_ is equal to 68 and thus *f*(THI) is equal to max (0, THI − 68).

### Statistical model

The following multitrait linear repeatability test‐day model was used for all the analyses, considering the first three parities as different traits as in Aguilar *et al. *(2009):

$$y_{\mathrm{klmn}}={\text{HTD}}_{\mathrm{kl}}+{\text{DIM}}_{m}+a_{\mathrm{nl}}+{\text{pe}}_{\mathrm{nl}}+v_{\mathrm{nl}}\left[f\left(\text{THI}\right)\right]+q_{\mathrm{nl}}\left[f\left(\text{THI}\right)\right]+e_{\mathrm{klmn}},$$

where *y_klmn_* is the outcome of the insemination (binary trait, 0 or 1), HTD*_kl_* is the fixed effect of herd‐day *k* of insemination within parity *l* (*l* = 1, 2, 3), DIM*_m_* is the *m*th DIM class of insemination with classes defined every 20 days, *a_nl_* is the general random additive genetic effect (intercept) of animal *n* in parity *l*, pe*_nl_* is the general random permanent environmental effect (intercept) of cow *n* in parity *l*, *f*(THI) is a function of THI for herd‐day *k* of insemination, *v_nl_* is the random regression additive genetic effect (slope) of conception per insemination per degree of THI above the threshold for the animal *n* in parity *l* (thermotolerance), *q_nl_* is the random regression permanent environmental effect (slope) of thermotolerance of the cow *n* in parity *l* and *e_klmn_* is the random residual effect.

Let $u=[a_{\mathrm{nl}}^{\prime }\;v_{\mathrm{nl}}^{\prime }]$ be a vector of random additive genetic effects and $p=[pe_{nl,}^{\prime }\;q_{\mathrm{nl}}^{\prime }]$ be a vector of random permanent environmental effects for lactations *n* = 1–3. The (co)variance structure was:

$$\text{Var}\left[\begin{array}{c} u\\ p\\ e \end{array}\right]=\left[\begin{array}{ccc} A⊗\Phi & 0 & 0\\ 0 & I⊗\psi & 0\\ 0 & 0 & I⊗R \end{array}\right],$$

where **A** is the numerator relationship matrix, **Φ** and **ψ** are 6 × 6 (co)variance matrices of random regression coefficients for additive and permanent environment effects respectively (three traits with two parameters, i.e. intercept and slope, per trait), **R** is a 3 × 3 diagonal matrix of residual variances corresponding to each trait and ⊗ denotes the Kronecker product of matrices.

### Genetic parameter estimation

Variance components were estimated using multitrait repeatability test‐day models in a Bayesian framework using the software gibbs2f90. Genotype data were not used for the estimation of variance components. Of a total of 500 000 samples, the first 100 000 samples were discarded as burn‐in, and every 100th sample was retained to calculate features of the posterior distributions, such as posterior means and standard deviations. Convergence diagnostics of Markov chain Monte Carlo sampling output were performed by visual inspection of trace plots.

Heritability (*h* ^2^) for conception per insemination at heat stress level *f*(*i*) was calculated as:

$$h^{2}=\frac{\sigma_{a}^{2}+f{\left(i\right)}^{2}\sigma_{v}^{2}+2f\left(i\right)\sigma_{\text{av}}}{\sigma_{a}^{2}+f{\left(i\right)}^{2}\sigma_{v}^{2}+2f\left(i\right)\sigma_{\text{av}}+\sigma_{\text{pe}}^{2}+f{\left(i\right)}^{2}\sigma_{q}^{2}+2f\left(i\right)\sigma_{\text{pq}}+\sigma_{e}^{2}},$$

where $\sigma_{a}^{2}$ is the variance of general additive genetic effects, $\sigma_{v}^{2}$ is the variance of thermotolerance additive genetic effects, $\sigma_{\text{av}}$ is the additive genetic covariance between general and thermotolerance effects, $\sigma_{\text{pe}}^{2}$ is the variance of general environmental permanent effects, $\sigma_{q}^{2}$ is the variance of thermotolerance environmental permanent effects, $\sigma_{\text{pq}}$ is the environmental permanent covariance between general and thermotolerance effects, *f*(*i*) is a function of THI and $\sigma_{e}^{2}$ is the residual variance. The genetic correlation between general and thermotolerance additive effects for conception per insemination was estimated as:

$$\text{corr}[a,\left.f(i)v\right]=\frac{f\left(i\right)\sigma_{\text{av}}}{\sqrt{\left(\sigma_{a}^{2}\cdot f{\left(i\right)}^{2}\sigma_{v}^{2}\right)}}.$$

### Gene mapping

The identification of genomic regions and individual genes affecting cow conception was performed using single‐step genomic BLUP (ssGBLUP). In ssGBLUP, the statistical model is the same as the classic BLUP, but the inverse of the pedigree relationship matrix (**A** ^−1^) is replaced with inverse of the realized relationship matrix (**H** ^−1^) that combines both pedigree and genomic information (Aguilar *et al.* 2010). The combined pedigree genomic relationship matrix **H** ^−1^ was calculated as follows:

$$H^{-1}=A^{-1}+\left[\begin{array}{cc} 0 & 0\\ 0 & G^{-1}-A_{22}^{-1} \end{array}\right]$$

where **G** ^−1^ is the inverse of the genomic relationship matrix and $A_{22}^{-1}$ is the inverse of the pedigree relationship matrix of the animals with genotype data. In this study, **G** ^−1^ has the dimension of 6292 × 6292 which includes 4700 cows with insemination records and 1592 sires in the pedigree. The **A** matrix has a dimension of 28 620 × 28 620, which was calculated based on a five‐generation pedigree.

Candidate regions and individual genes associated with cow conception under thermo‐neutral and thermo‐stress conditions were identified based on the amount of genetic variance explained by 2.0 Mb windows of adjacent SNPs. Given the vector of genomic estimated breeding values, the SNP effects can be estimated as $\hat{s}=DZ^{\prime }{\left[ZDZ^{\prime }\right]}^{-1}\hat{u}$, where $\hat{s}$ is the vector of SNP marker effects, **D** is a diagonal matrix of weight of SNPs (in this study, **D** was set as an identity matrix), **Z** is a matrix relating genotypes of each SNP marker to observations and $\hat{u}$ is the vector of genomic estimated breeding values (Wang *et al.* 2012). The percentage of genetic variance explained by a given 2.0 Mb window of adjacent SNPs was then calculated as:

$$\frac{\text{var}(u_{i})}{\sigma_{u}^{2}}\times 100=\frac{\text{var}\left(\sum_{j=1}^{B}z_{j}s_{j}\right)}{\sigma_{u}^{2}}\times 100,$$

where *u_i_* is the genetic value of the *i*th genomic region under consideration, *B* is the total number of adjacent SNPs within 2.0 Mb region, *z_j_* is the genotype code of *j*th marker and *s_j_* is the marker effect of the *j*th SNP within the *i*th region. All of these calculations were performed using postgsf90 of the blupf90 family of programs (Aguilar *et al.* 2014).

### Gene‐set analysis

Gene‐set enrichment analysis, also known as overrepresentation or pathway analysis, is a powerful tool that allows us to reveal biological pathways and molecular mechanisms underlying phenotypes of interest. Here, we conducted alternative gene‐set analyses using different annotation databases, including GO, Medical Subject Headings, InterPro and Reactome, in order to obtain additional insights regarding biological processes that might explain cows’ ability to conceive under thermal stress conditions. The gene‐set analysis consisted basically of three steps (Han & Peñagaricano 2016): first, the assignment of SNP markers to annotated genes; then the assignment of genes to functional gene‐sets or pathways; and finally the association between each functional term and the phenotype of interest.

The UMD 3.1 bovine genome sequence assembly was used for SNP assignment using bioconductor r package biomart (Durinck *et al.* 2009). Here, SNPs were assigned to genes if they were located within the genomic sequence of the gene or at most 15 kb either upstream or downstream of the gene. The distance of 15 kb was chosen to capture regulatory regions that may lie close to but outside of the gene. An arbitrary threshold of 5% of the SNP effects distribution (in absolute value) was used to define relevant SNP markers, and potential thermotolerant genes were defined as those genes flagged by at least one relevant SNP. Finally, the identification of gene‐sets or pathways significantly enriched with thermotolerant genes was performed using a Fisher’s exact test, a test of proportions based on the cumulative hypergeometric distribution (Peñagaricano *et al.* 2012).

## Results and discussion

### Genetic parameter estimation

Variance components for conception per insemination under thermo‐neutral (intercept) and thermo‐stress (slope) conditions were estimated using multitrait linear repeatability test day models (Table 1). Relevant genetic parameters include heritability and genetic correlations at THI = 78, i.e. moderate heat stress level, across the first three parities. Additive genetic variances under heat stress conditions increased between 80 and 200% across the first three parities, suggesting that heat stress has greater effects on conception rate in later lactations. Similarly, Biffani *et al. *(2016) reported that additive genetic variances for 56‐day non‐return rate under heat stress conditions increased from first to second parity in Italian Holstein cows. The increase in thermotolerance additive genetic variances across parities suggests that multiparous cows are more susceptible than primiparous cows to the negative effects of heat stress on reproductive performance.

**Table 1:** General ($\sigma_{a}^{2}$) and thermotolerance ($100\;\sigma_{v}^{2}$) additive genetic variances, genetic correlations ($r_{a,v}^{G}$) and heritability ($h_{f(10)}^{2}$) at temperature–humidity index = 78.

Parameters	Parity 1	Parity 2	Parity 3
$\sigma_{a}^{2}$	0.002	0.004	0.008
$100\;\sigma_{v}^{2}$	0.005	0.009	0.015
10 σ_av_	−0.001	−0.003	−0.008
$\sigma_{e}^{2}$	0.20	0.19	0.18
$h_{f(10)}^{2}$ (95% HPD)	0.024 (0.009, 0.039)	0.032 (0.013, 0.051)	0.024 (0.007, 0.048)
$r_{\text{a,v}}^{G}$ (95% HPD)	−0.35 (−0.73, 0.07)	−0.58 (−0.84, −0.34)	−0.82 (−0.95, −0.67)
cor_ht_ (par_1_, par_j_)		0.17	0.49
cor_ht_ (par_2_, par_3_)			0.32
cor_gen_ (par_1_, par_j_)		0.58	0.83
cor_gen_ (par_2_, par_3_)			0.88

HPD, Highest posterior density; $\sigma_{a}^{2}$, general additive genetic variance; $\sigma_{e}^{2}$, residual variance; $100\;\sigma_{v}^{2}$, thermotolerance additive genetic variance; 10 σ_av_, additive genetic covariance between general and thermotolerance effect; $r_{\text{a,v}}^{G}$, genetic correlation between general and thermotolerance effect; cor_ht_, thermotolerance additive genetic correlation; and cor_gen_, general additive genetic correlation.

Heritability estimates for conception per insemination at THI = 78, i.e. *f*(THI) = 10, were between 2 and 3% across the first three parities. Our findings are similar to those reported by Ravagnolo & Misztal (2002), who estimated a heritability equal to 1.4% for non‐return rate 60 days after insemination at THI = 70 in primiparous US Holstein cows. Similarly, Hagiya *et al. *(2017) reported heritability estimates for conception rate between 1 and 3% in Holstein cattle in Japan under both mild and moderate heat stress conditions. Biffani *et al. *(2016) reported heritability estimates for 56‐day non‐return rate under heat stress of between 2.5 and 7.6% for the first two parities in Italian Holstein cows. Overall, all these studies suggest that there is a significant genetic variability underlying cow conception under heat stress, and therefore, genetic selection for improved fertility under thermal stress conditions is feasible.

Genetic correlations between general and thermotolerance additive genetic effects were always negative and ranged from −0.35 to −0.82 across the first three parities. These negative genetic correlations suggest that the continued selection for greater cow fertility under thermo‐neutral conditions will result in increasing even more the harmful effects of heat stress on reproductive performance. Our results are in agreement with the findings of several previous studies. For instance, for 56‐day non‐return rate in Italian Holstein cows, genetic correlations between general and thermotolerance stress effects were −0.31 for first‐parity cows and −0.45 for second‐parity cows (Biffani *et al.* 2016). Similarly, first‐parity US Holstein cows showed a marked negative genetic correlation (−0.77) for 60‐day non‐return rate between regular and heat tolerance effects (Ravagnolo & Misztal 2002). The negative relationship between cows’ ability to conceive under thermo‐neutral vs. thermo‐stress conditions emphasize the importance of selecting animals in the same environment under which they are going to reproduce.

### Gene mapping

**Figure 1:**
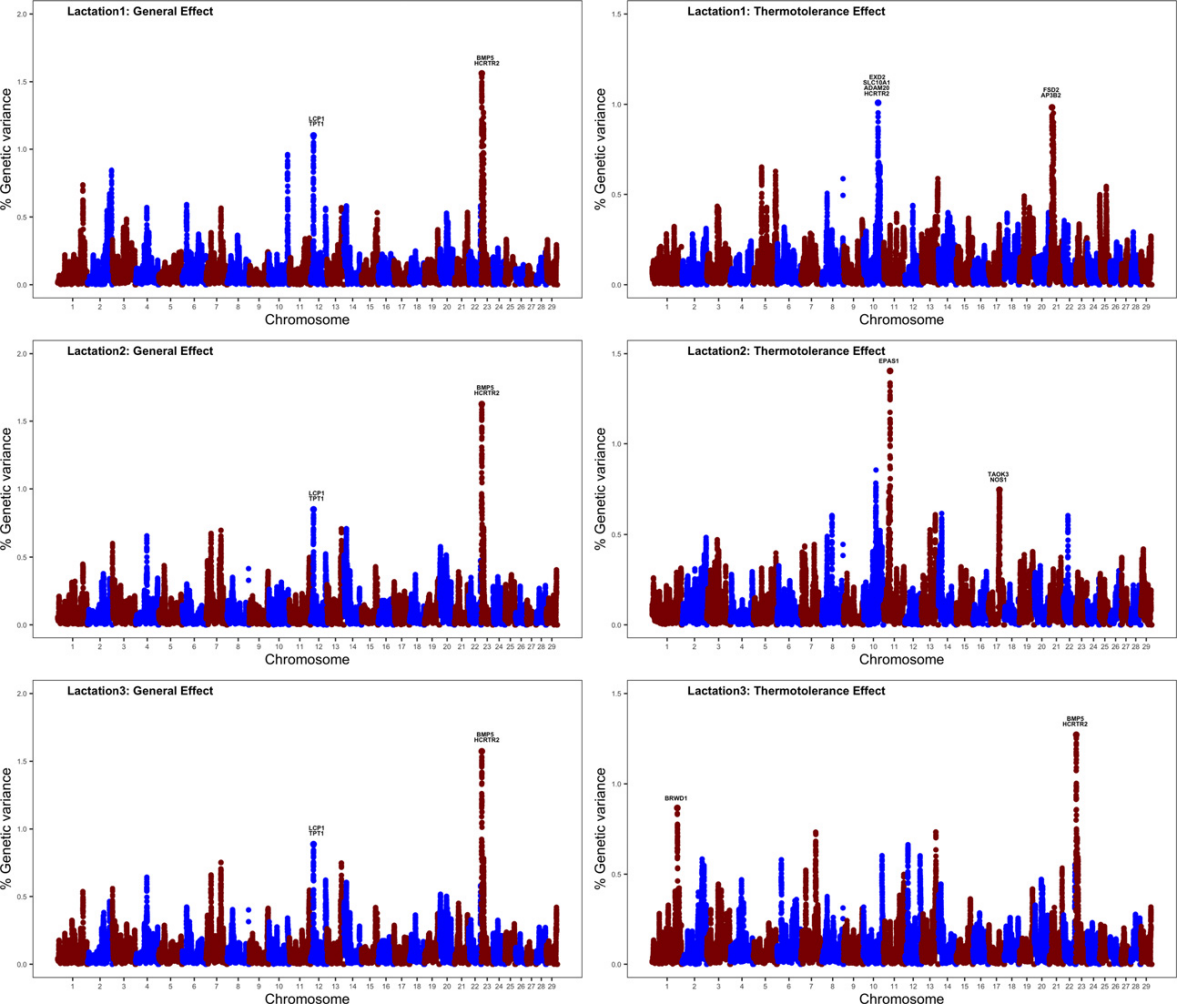
Whole‐genome scans for conception per insemination for the first three parities (numbered vertically as lactation 1, lactation 2 and lactation 3). The left plots highlight genomic regions and candidate genes affecting cow conception under thermo‐neutral conditions (general additive genetic effects), whereas the right plots highlight genomic regions and putative genes implicated in cow conception under heat stress conditions (thermotolerance additive genetic effects).

Figure 1 displays the results of the genomic scans for conception per insemination across the first three lactations under study. The results are presented in terms of the proportion of the additive genetic variance explained by 2.0 Mb SNP windows. Left plots report the genomic regions associated with cow conception under thermo‐neutral conditions, whereas the right plots report the genomic regions affecting cow conception under heat stress.

Genomic scans under thermo‐neutral conditions clearly identified a peak on BTA23 at 3.60–5.60 Mb that explains more than 1.5% of additive genetic variance across the three parities. This genomic region harbors at least two putative genes, *BMP5* and *HCRTR2*, that are directly implicated in early embryo development. Gene *BMP5* encodes a member of the transforming growth factor‐*β* superfamily and is highly expressed in placenta. Interestingly, previous research has shown that early addition of BMP5 to the embryo culture medium had a positive effect on the blastocyst rate, affecting the expression of several BMP target and pluripotency genes, suggesting that *BMP5* plays an important role in the preimplantation development of bovine embryos (Garcia *et al.* 2015). Gene *HCRTR2* encodes a G‐protein coupled receptor involved in the regulation of feeding behavior and energy metabolism that appears to be directly implicated in uterine processes during early pregnancy (Smolinska *et al.* 2017). Another 2.0 Mb SNP window located on BTA12 at 13.48–13.66 Mb also explained a significant amount of general genetic variance across all three lactations. This region harbors at least two genes, *LCP1* and *TPT1*, which are related to embryonic development. Gene *LCP1* is involved in gastrulation and embryonic epidermal development (Baumgartner *et al.* 2019). Gene *TPT1* is expressed in placenta and is implicated in calcium binding and homeostasis of trophoblast cells, which is important for growth and development of the fetus (Arcuri *et al.* 2005).

The ssGBLUP method also revealed several genomic regions and putative genes that partly explain the observed variation in cow conception under heat stress conditions (Fig. 1, right plots). Interestingly, most of the regions that explained a large amount of thermotolerance additive genetic variance are parity specific. For primiparous cows, the two most important regions are located on BTA10 (80.75–82.75 Mb) and BTA21 (21.31–23.31 Mb). The genomic region on BTA10 harbors at least two genes, *EXD2* and *SLC10A1*, that are involved in cellular response to heat stress. Gene *EXD2* is implicated in the cellular response to oxidative stress owing to heat stress; it protects the cells from oxidative stress by reducing reactive oxygen species (ROS) production. Indeed, the KO of *EXD2* results in oxidative stress and cell death (Zlotorynski 2018). Gene *SLC10A1* is involved in cholesterol biosynthesis and metabolism, and cholesterol plays an important role in cellular protection against heat stress through the activation of heat shock proteins production (Balogh *et al.* 2013). In addition, gene *ADAM20*, also located on BTA10 at 80.75–82.75 Mb, encodes a membrane‐anchored protein involved in cell–cell and cell–matrix interactions, including fertilization and early embryonic development (Sha *et al.* 2018). Moreover, the genomic region on BTA21 at 21.31–23.31 Mb harbors at least two relevant genes, *FSD2* and *AP3B2.* Gene *FSD2* is an oxidative stress‐related gene which acts as an ROS scavenger, protecting the cells from oxidative damage caused by heat stress (Myouga *et al.* 2008). Gene *AP3B2* is implicated in the intracellular removal of heat‐induced protein aggregates, maintaining protein homeostasis (Wilbe *et al.* 2015).

For second‐lactation cows, two different regions on BTA11 (27.18–29.18 Mb) and BTA17 (56.50–58.50 Mb) explained large amounts of additive genetic variance for conception per insemination under heat stress. The genomic region on BTA11, which explained almost 1.5% of thermotolerance genetic variance, harbors gene *EPAS1*, which is an oxidative stress‐related gene, directly implicated the hypoxic response, a common condition during thermal stress. The KO of *EPAS1* in mice results in early embryonic mortality because of hypoxia (Xu *et al.* 2014). Notably, gene *EPAS1* has already been associated with both fertility traits and rectal temperature/thermotolerance in dairy cattle (Dikmen *et al.* 2015; Ortega *et al.* 2016). Moreover, the genomic region on BTA17 at 56.50–58.50 Mb harbors at least two genes, namely *TAOK3* and *NOS1*, that are directly implicated in the cellular response to heat stress. In fact, gene *TAOK3* is involved in the repair of DNA owing to different insults that damage DNA such as thermal stress. The knockdown of *TAOK3* inhibits the cellular response to DNA damage, resulting in cell death (Raman *et al.* 2007). Gene *NOS1* is implicated in the maintenance of cellular redox homeostasis owing to its ability to neutralize harmful ROS produced during heat stress. Gene *NOS1* enhances the antioxidant enzyme activities of glutathione reductase and ascorbate peroxidase, which have a protective role during heat stress (Shi *et al.* 2014).

The genomic region located on BTA1 at 139.09–141.00 Mb explained almost 1% of the additive genetic variance for conception per insemination under heat stress for third parity. Notably, this region harbors gene *BRWD1*, which is involved in meiotic chromosomal stability in the oocytes, and hence plays a crucial role in ensuring a successful conception (Pattabiraman *et al.* 2015).

Overall, the whole‐genome scans identified several regions implicated in dairy cow conception under heat stress conditions. Interestingly, most of these genomic regions harbor candidate genes that are directly involved in either the cellular response to heat stress and/or fertilization and early embryo development. Table S1 provides information about putative functional variants that are polymorphic in Holstein cows and are located either within or close to (±5 kb) these candidate genes. This information was kindly provided by the 1000 Bull Genomes Project (http://www.1000bullgenomes.com) based on whole‐genome sequence data from 824 Holstein bulls.

### Over‐representation analysis

Of the 58 046 SNP markers evaluated in the whole‐genome association mapping, 27 488 SNPs were located either within or near annotated genes. This set of SNPs defined a total of 19 305 genes annotated in the bovine reference genome. A subset of 928 of these 19 305 genes were flagged by at least one relevant SNP (top 5% of the SNP effects distribution) in at least two parities, and hence these 928 genes were defined as relevant genes for cow conception under heat stress conditions.

**Figure 2:**
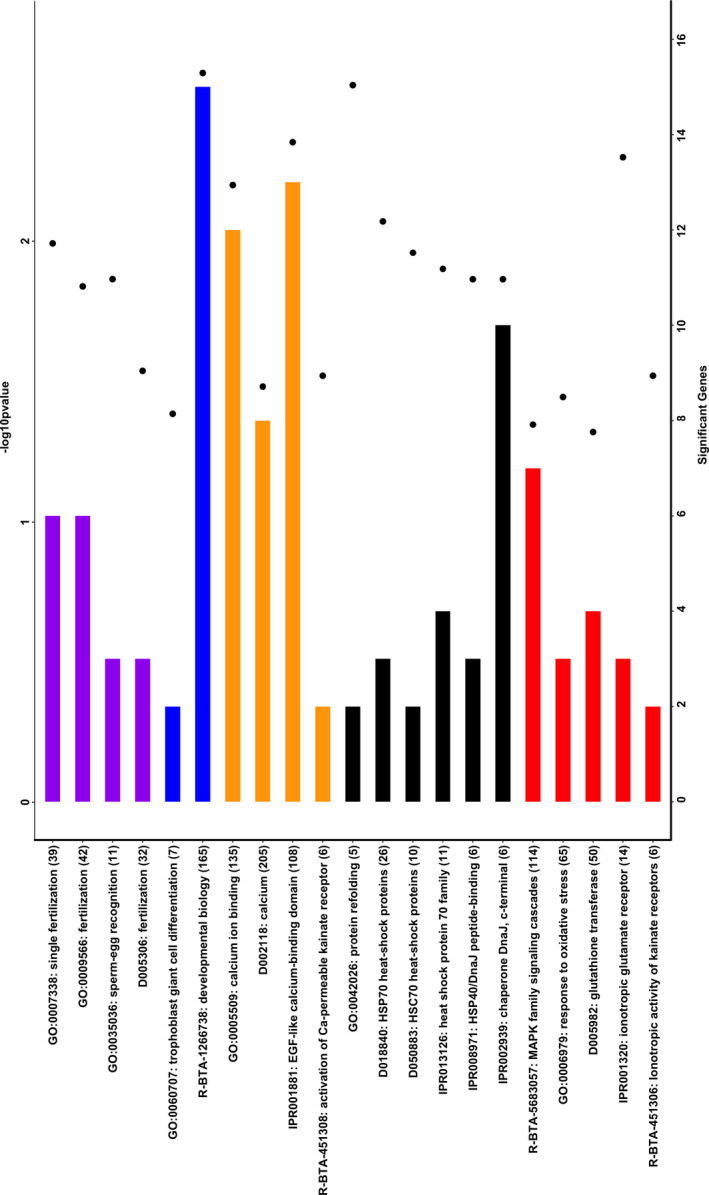
Functional terms and pathways significantly enriched with genes associated with dairy cow conception under thermal‐stress conditions. Four gene annotation databases were analyzed: GO, Medical Subject Headings, InterPro and Reactome. The *y*‐axis displays the names and the total number of genes of each gene‐set. The black dots represent the significance of enrichment (−log_10_ *P*‐value, Fisher’s exact test, top *x*‐axis) and the bars represent the number of significant genes in each functional term (bottom *x*‐axis).

Figure 2 shows the most relevant functional terms and biological pathways enriched with thermotolerant genes. Note that the over‐representation analysis interrogated four different databases, GO, Medical Subject Headings, InterPro and Reactome.

At least five different groups of gene‐sets were identified; these functional terms are related to fertilization, development, heat shock proteins, cellular response to oxidative stress, and calcium ion homeostasis. Table S2 reports the full list of significant GO terms, including GO ID, GO name, total number of genes, number of relevant genes and Fisher’s *P*‐value.

Noticeably, some of most significant terms are directly involved in cow conception and early embryo development, such as *fertilization* (D005306), *sperm‐egg recognition* (GO:0035036), *single fertilization* (GO:0007388), *trophoblast giant cell differentiation* (GO:0060707) and *developmental biology* (R‐BTA‐1266738). These significant reproductive terms had in common at least six thermotolerant genes, namely *SPESP1*, *PLCZ1*, *HAND1*, *FYN*, *DOCK1* and *PI*. All of these genes are involved in the process of fertilization and subsequent early embryo development (Halet *et al.* 2008; Fujihara *et al.* 2010).

Of special interest, many gene‐sets that showed an overrepresentation of thermotolerant genes are directly associated with cellular response to heat stress, such as *protein refolding* (GO:0042026), *HSP70 heat‐shock proteins* (D018840), *heat shock protein 70 family* (IPR013126), *HSP40/DnaJ peptide‐binding* (IPR008971), *HSC70 heat‐shock proteins* (D050883) and *chaperone DnaJ, c‐terminal* (IPR002939). These heat shock response terms contain at least four relevant genes, namely *HSF1*, *DNAJB4*, *CAMK2D* and *HSPA1A*, all of them directly involved in cellular response to heat stress by maintaining proper protein folding, preserving cytoskeletal integrity and removing misfolded proteins during thermal stress (Collier *et al.* 2008; Gray & Heller Brown 2014; Min *et al.* 2015).

There are also some relevant functional terms related to oxidative stress, including *response to oxidative stress* (GO:0006979), *glutathione transferase* (D005982), *ionotropic glutamate receptor* (IPR001320) and *ionotropic activity of kainate receptors* (R‐BTA‐451306). Oxidative stress occurs as a consequence of an imbalance between ROS production and the antioxidant defense, and heat stress is an environmental factor responsible for stimulating ROS production. These terms were significantly enriched with genes *GRIK2*, *PRDX1* and *PSEN1*, all directly implicated in cell survival during heat stress.

Calcium ion homeostasis and metabolism was also identified as a biological process significantly enriched with genes implicated in cow conception under heat stress conditions. Indeed, terms such as *calcium ion binding* (GO:0005509), *calcium* (D002118), *EGF‐like calcium binding domain* (IPR001881) and *activation of Ca‐permeable kainite receptor* (R‐BTA‐451308) were among the most significant ones. These calcium‐related gene‐sets contain at least three relevant genes, *MYL2*, *NELL2* and *PLCZ1*, that are associated with fertilization and embryonic development.

## Conclusions

In this study, we performed a comprehensive genetic and genomic analysis in order to reveal the genetic and biological basis of reproductive performance of dairy cows under heat stress conditions. Heritability estimates for conception per insemination under heat stress ranged between 2 and 3%, suggesting that the ability of a dairy cow to conceive under heat stress is influenced by genetic factors, and hence it could be improved by genetic means. Notably, the genetic correlations between general and thermotolerance additive genetic effects were negative and ranged from −0.35 to −0.82. These findings reinforce the idea that there is a negative relationship between cows’ ability to conceive under thermo‐neutral vs. thermo‐stress conditions, and hence the continued selection for greater fertility ignoring heat tolerance will result in even greater increases in susceptibility to heat stress. Therefore, the dairy industry should focus on selecting cows that conceive well regardless of the level of heat stress. The whole‐genome scans identified at least six regions located on BTA1, BTA10, BTA11, BTA17, BTA21 and BTA23 that explained a large amount of thermotolerance additive genetic variances. Interestingly, most of these regions harbor genes directly implicated in either fertilization and early embryo development or cellular response to heat stress. The gene‐set analysis revealed at least five relevant processes, namely fertilization and development, protein misfolding and heat shock proteins, cellular response to oxidative stress and calcium ion homeostasis, as significantly enriched with genes associated with cow conception under heat stress. Overall, this study contributes to a better, deeper understanding of the effect that heat stress has on cow reproduction. Our findings may also contribute to the development of novel breeding strategies to improve cow reproductive performance under heat stress.

## Supporting information


**Table S1** List of genetic variants segregating within or close to (±5 kb) candidate genes.

**Table S2** List of significant gene‐sets.

## Data Availability

Phenotypic and genotypic data were obtained from North Florida Holsteins (Bell, FL, USA), the University of Florida Dairy Research Unit (Alachua, FL, USA), the Dairy Record Management System (Raleigh, NC, USA) and the Council on Dairy Cattle Breeding (Bowie, MD, USA). These datasets were used under material transfer agreement, and hence, are not publicly available. However, data are available upon request to FP and with the permission of North Florida Holsteins, University of Florida Dairy Research Unit, and Cooperative Dairy DNA Repository.

## References

[age12943-bib-0001] Aguilar I. , Misztal I. & Tsuruta S. (2009) Genetic components of heat stress for dairy cattle with multiple lactations. Journal of Dairy Science 92, 5702–11. 19841230 10.3168/jds.2008-1928

[age12943-bib-0002] Aguilar I. , Misztal I. , Johnson D.L. , Legarra A. , Tsuruta S. & Lawlor TJ (2010) Hot topic: a unified approach to utilize phenotypic, full pedigree, and genomic information for genetic evaluation of Holstein final score. Journal of Dairy Science 93, 743–52. 20105546 10.3168/jds.2009-2730

[age12943-bib-0003] Aguilar I. , Misztal I. , Tsuruta S. , Legarra A. & Wang H. (2014) PREGSF90–POSTGSF90: computational tools for the implementation of single‐step genomic selection and genome‐wide association with ungenotyped individuals in BLUPF90 programs In: Proceedings of the 10th World Congress of Genetics Applied to Livestock Production. Vancouver, Canada.

[age12943-bib-0004] Al‐Katanani Y.M. , Paula‐Lopes F.F. & Hansen P.J. (2002) Effect of season and exposure to heat stress on oocyte competence in Holstein cows. Journal of Dairy Science 85, 390–6. 11913699 10.3168/jds.s0022-0302(02)74086-1

[age12943-bib-0005] Ansari‐Mahyari S. , Ojali M.R. , Forutan M. , Riasi A. & Brito L.F. (2019) Investigating the genetic architecture of conception and non‐return rates in Holstein cattle under heat stress conditions. Tropical Animal Health and Production 51, 1847–53. 30941706 10.1007/s11250-019-01875-5

[age12943-bib-0006] Arcuri F. , Papa S. , Meini A. *et al* (2005) The translationally controlled tumor protein is a novel calcium binding protein of the human placenta and regulates calcium handling in trophoblast cells. Biology of Reproduction 73, 745–51. 15958728 10.1095/biolreprod.105.042077

[age12943-bib-0007] Averill T.A. , Rekaya R. & Weigel K. (2004) Genetic analysis of male and female fertility using longitudinal binary data. Journal of Dairy Science 87, 3947–52. 15483179 10.3168/jds.S0022-0302(04)73534-1

[age12943-bib-0008] Balogh G. , Peter M. , Glatz A. , Gombos I. , Torok Z. , Horvath I. , Harwood J.L. & Vigh L. (2013) Key role of lipids in heat stress management. Febs Letters 587, 1970–80. 23684645 10.1016/j.febslet.2013.05.016

[age12943-bib-0009] Baumgartner E.A. , Compton Z.J. , Evans S. , Topczewski J. & LeClair E.E. (2019) Identification of regulatory elements recapitulating early expression of L‐plastin in the zebrafish enveloping layer and embryonic periderm. Gene Expression Patterns 32, 53–66. 30940554 10.1016/j.gep.2019.03.001 PMC6655599

[age12943-bib-0010] Biffani S. , Bernabucci U. , Vitali A. , Lacetera N. & Nardone A. (2016) Short communication: effect of heat stress on nonreturn rate of Italian Holstein cows. Journal of Dairy Science 99, 5837–43. 27108174 10.3168/jds.2015-10491

[age12943-bib-0011] Bohmanova J. , Misztal I. & Cole J.B. (2007) Temperature‐humidity indices as indicators of milk production losses due to heat stress. Journal of Dairy Science 90, 1947–56. 17369235 10.3168/jds.2006-513

[age12943-bib-0012] Collier R.J. , Collier J.L. , Rhoads R.P. & Baumgard L.H. (2008) Invited review: genes involved in the bovine heat stress response. Journal of Dairy Science 91, 445–54. 18218730 10.3168/jds.2007-0540

[age12943-bib-0013] Dikmen S. , Wang X.Z. , Ortega M.S. , Cole J.B. , Null D.J. & Hansen P.J. (2015) Single nucleotide polymorphisms associated with thermoregulation in lactating dairy cows exposed to heat stress. Journal of Animal Breeding and Genetics 132, 409–19. 26198991 10.1111/jbg.12176

[age12943-bib-0014] Durinck S. , Spellman P.T. , Birney E. & Huber W. (2009) Mapping identifiers for the integration of genomic datasets with the R/Bioconductor package biomaRt. Nature Protocols 4, 1184–91. 19617889 10.1038/nprot.2009.97 PMC3159387

[age12943-bib-0015] Fujihara Y. , Murakami M. , Inoue N. , Satouh Y.. , Kaseda K. , Ikawa M. & Okabe M. (2010) Sperm equatorial segment protein 1, SPESP1, is required for fully fertile sperm in mouse. Journal of Cell Science 123, 1531–6. 20375058 10.1242/jcs.067363

[age12943-bib-0016] Garcia E.V. , Miceli D.C. , Rizo G.. , Valdecantos P.A. & Barrera A.D. (2015) Effect of early addition of bone morphogenetic protein 5 (BMP5) to embryo culture medium on in vitro development and expression of developmentally important genes in bovine preimplantation embryos. Theriogenology 84, 589–99. 26014926 10.1016/j.theriogenology.2015.04.018

[age12943-bib-0017] Gray C.B. & Heller Brown J. (2014) CaMKIIdelta subtypes: localization and function. Front Pharmacol 5, 15. 24575042 10.3389/fphar.2014.00015 PMC3920101

[age12943-bib-0018] Hagiya K. , Hayasaka K. , Yamazaki T. , Shirai T. , Osawa T. , Terawaki Y. , Nagamine Y. , Masuda Y. & Suzuki M. (2017) Effects of heat stress on production, somatic cell score and conception rate in Holsteins. Animal Science Journal 88, 3–10. 27113198 10.1111/asj.12617

[age12943-bib-0019] Halet G. , Viard P. & Carroll J. (2008) Constitutive PtdIns(3,4,5)P3 synthesis promotes the development and survival of early mammalian embryos. Development 135, 425–9. 18094023 10.1242/dev.014894

[age12943-bib-0020] Han Y. & Peñagaricano F. (2016) Unravelling the genomic architecture of bull fertility in Holstein cattle. BMC Genetics 17, 143. 27842509 10.1186/s12863-016-0454-6 PMC5109745

[age12943-bib-0021] Hansen P.J. (2009) Effects of heat stress on mammalian reproduction. Philosophical Transactions of the Royal Society of London. Series B, Biological Sciences 364, 3341–50. 19833646 10.1098/rstb.2009.0131 PMC2781849

[age12943-bib-0022] Hansen P.J. & Arechiga C.F. (1999) Strategies for managing reproduction in the heat‐stressed dairy cow. Journal of Animal Science 77, 36–50. 15526779 10.2527/1997.77suppl_236x

[age12943-bib-0023] Hoglund J.K. , Guldbrandtsen B. , Lund M.S. & Sahana G. (2015) Identification of genomic regions associated with female fertility in Danish Jersey using whole genome sequence data. BMC Genetics 16, 60. 26036962 10.1186/s12863-015-0210-3 PMC4453229

[age12943-bib-0024] Jordan E.R. (2003) Effects of heat stress on reproduction. Journal of Dairy Science 86, E104–E114.

[age12943-bib-0025] Kiser J.N. , Clancey E. , Moraes J.G.N. , Dalton J. , Burns G.W. , Spencer T.E. & Neibergs H.L. (2019) Identification of loci associated with conception rate in primiparous Holstein cows. BMC Genomics 20, 840. 31718557 10.1186/s12864-019-6203-2 PMC6852976

[age12943-bib-0026] Liu A.X. , Wang Y.C. , Sahana G. , Zhang Q. , Liu L. , Lund M.S. & Su G.S. (2017) Genome‐wide association studies for female fertility traits in Chinese and Nordic Holsteins. Scientific Reports 7, 8487. 28814769 10.1038/s41598-017-09170-9 PMC5559619

[age12943-bib-0027] Min L. , Cheng J.B. , Shi B.L. , Yang H.J. , Zheng N. & Wang J.Q. (2015) Effects of heat stress on serum insulin, adipokines, AMP‐activated protein kinase, and heat shock signal molecules in dairy cows. Journal of Zhejiang University‐Science B 16, 541–8. 26055916 10.1631/jzus.B1400341 PMC4471606

[age12943-bib-0028] Myouga F. , Hosoda C. , Umezawa T. , Iizumi H. , Kuromori T. , Motohashi R. , Shono Y. , Nagata N. , Ikeuchi M. & Shinozaki K. (2008) A heterocomplex of iron superoxide dismutases defends chloroplast nucleoids against oxidative stress and is essential for chloroplast development in arabidopsis. Plant Cell 20, 3148–62. 18996978 10.1105/tpc.108.061341 PMC2613658

[age12943-bib-0029] NOAA (1976) Livestock hot weather stress In: Operations Manual Letter C‐31‐76. United States Department of Commerce, National Oceanic and Atmospheric Administration, Kansas City, MO.

[age12943-bib-0030] Ortega M.S. , Denicol A.C. , Cole J.B. , Null D.J. & Hansen P.J. (2016) Use of single nucleotide polymorphisms in candidate genes associated with daughter pregnancy rate for prediction of genetic merit for reproduction in Holstein cows. Animal Genetics 47, 288–97. 26923315 10.1111/age.12420

[age12943-bib-0031] Pattabiraman S. , Baumann C. , Guisado D. , Eppig J.J. , Schimenti J.C. & De la Fuente R. (2015) Mouse BRWD1 is critical for spermatid postmeiotic transcription and female meiotic chromosome stability. Journal of Cell Biology 208, 53–69. 25547156 10.1083/jcb.201404109 PMC4284233

[age12943-bib-0032] Peñagaricano F. , Weigel K.A. , Rosa G.J. & Khatib H. (2012) Inferring quantitative trait pathways associated with bull fertility from a genome‐wide association study. Frontiers in Genetics 3, 307. 23335935 10.3389/fgene.2012.00307 PMC3542705

[age12943-bib-0033] Raman M. , Earnest S. , Zhang K. , Zhao Y. & Cobb M.H. (2007) TAO kinases mediate activation of p38 in response to DNA damage. EMBO Journal 26, 2005–14. 17396146 10.1038/sj.emboj.7601668 PMC1852793

[age12943-bib-0034] Ravagnolo O. & Misztal I. (2000) Genetic component of heat stress in dairy cattle, parameter estimation. Journal of Dairy Science 83, 2126–30. 11003247 10.3168/jds.S0022-0302(00)75095-8

[age12943-bib-0035] Ravagnolo O. & Misztal I. (2002) Effect of heat stress on nonreturn rate in Holstein cows: genetic analyses. Journal of Dairy Science 85, 3092–100. 12487476 10.3168/jds.S0022-0302(02)74396-8

[age12943-bib-0036] Santana M.L. , Bignardi A.B. , Pereira R.J. , Stefani G. & El Faro L. (2017) Genetics of heat tolerance for milk yield and quality in Holsteins. Animal 11, 4–14. 27532229 10.1017/S1751731116001725

[age12943-bib-0037] Sartori R. , Sartor‐Bergfelt R. , Mertens S.A. , Guenther J.N. , Parrish J.J. & Wiltbank M.C. (2002) Fertilization and early embryonic development in heifers and lactating cows in summer and lactating and dry cows in winter. Journal of Dairy Science 85, 2803–12. 12487447 10.3168/jds.S0022-0302(02)74367-1

[age12943-bib-0038] Schuller L.K. , Burfeind O. & Heuwieser W. (2014) Impact of heat stress on conception rate of dairy cows in the moderate climate considering different temperature‐humidity index thresholds, periods relative to breeding, and heat load indices. Theriogenology 81, 1050–7. 24612695 10.1016/j.theriogenology.2014.01.029

[age12943-bib-0039] Sha Y.W. , Xu X. , Ji Z.Y. , Mei L.B. , Qiu P.P. , Ji H. , Li P. , Li L. & Liu W.W. (2018) Sperm‐egg fusion disorder in a Chinese male patient was associated with a rare ADAM20 variant. Oncotarget 9, 2086–91. 29416755 10.18632/oncotarget.23331 PMC5788623

[age12943-bib-0040] Shi H. , Ye T. , Zhu J.K. & Chan Z. (2014) Constitutive production of nitric oxide leads to enhanced drought stress resistance and extensive transcriptional reprogramming in Arabidopsis. Journal of Experimental Botany 65, 4119–31. 24868034 10.1093/jxb/eru184 PMC4112625

[age12943-bib-0041] Sigdel A. , Abdollahi‐Arpanahi R. , Aguilar I. & Peñagaricano F. (2019) Whole genome mapping reveals novel genes and pathways involved in milk production under heat stress in US Holstein cows. Frontiers in Genetics 10, 928. 31636656 10.3389/fgene.2019.00928 PMC6788456

[age12943-bib-0042] Silva C.F. , Sartorelli E.S. , Castilho A.C. , Satrapa R.A. , Puelker R.Z. , Razza E.M. , Ticianelli J.S. , Eduardo H.P. , Loureiro B. & Barros C.M. (2013) Effects of heat stress on development, quality and survival of *Bos indicus* and *Bos taurus* embryos produced in vitro. Theriogenology 79, 351–7. 23154141 10.1016/j.theriogenology.2012.10.003

[age12943-bib-0043] Smolinska N. , Kiezun M. , Dobrzyn K. , Szeszko K. , Maleszka A. & Kaminski T. (2017) Adiponectin, orexin A and orexin B concentrations in the serum and uterine luminal fluid during early pregnancy of pigs. Animal Reproduction Science 178, 5–12. 10.1016/j.anireprosci.2017.01.001 28089263

[age12943-bib-0044] De Vries A. (2006) Economic value of pregnancy in dairy cattle. Journal of Dairy Science 89, 3876–85. 16960063 10.3168/jds.S0022-0302(06)72430-4

[age12943-bib-0045] de Vries A. & Risco C.A. (2005) Trends and seasonality of reproductive performance in Florida and Georgia dairy herds from 1976 to 2002. Journal of Dairy Science 88, 3155–65. 16107406 10.3168/jds.S0022-0302(05)72999-4

[age12943-bib-0046] Wang H. , Misztal I. , Aguilar I. , Legarra A. & Muir W.M. (2012) Genome‐wide association mapping including phenotypes from relatives without genotypes. Genetics Research 94, 73–83. 22624567 10.1017/S0016672312000274

[age12943-bib-0047] Wilbe M. , Kozyrev S.V. , Farias F.H.G. *et al* (2015) Multiple changes of gene expression and function reveal genomic and phenotypic complexity in SLE‐like disease. PLoS Genetics 11, e1005248. 26057447 10.1371/journal.pgen.1005248 PMC4461293

[age12943-bib-0048] Xu X.H. , Huang X.W. , Qun L. *et al* (2014) Two functional loci in the promoter of EPAS1 gene involved in high‐altitude adaptation of Tibetans. Scientific Reports 4, 7465. 25501874 10.1038/srep07465 PMC4264014

[age12943-bib-0049] Zlotorynski E. (2018) Mechanisms of disease: Consequences of mitoribosome overload. Nature Reviews Molecular Cell Biology 19, 140–1. 10.1038/nrm.2018.8 29382939

